# Supplementary material for: Ion-dependent structure, dynamics, and allosteric coupling in a non-selective cation channel
Source: Nat Commun. 2021 Oct 28;12:6225. doi: 10.1038/s41467-021-26538-8 (PMC8553846; doi:10.1038/s41467-021-26538-8)
Supplement: Supplementary file 1 — Supplementary Information [file 41467_2021_26538_MOESM1_ESM.pdf]

Supplementary note 1: SDS-mediated back-exchange enables solution NMR studies of NaK

As noted in the text, SDS-mediated refolding and back-exchange greatly increased the number of peaks visible in perdeuterated NaK samples (Fig. S1). For K^+, most residues are at least partially back exchanged, enabling assignment of the majority of peaks in SDS back-exchanged spectra. Assignments were also aided by previous 3D experiments collected at 60ºC on non-deuterated samples where more signals are present, albeit with lower s/n than for deuterated samples. However there are four peaks that appear that remain unassigned, as 3D experiments were not performed on back-exchanged samples. It is likely that these peaks correspond to the four unassigned residues, 57, 60, 61, and 63. Notably, the peak observed for T62 and one of the new peaks that appear are quite weak, suggesting that there could be intermediate timescale dynamics occurring in this region or that H/D exchange is not complete in this region even in SDS. In the case of Na^+, as well, we lack assignments for residues 57 and 60-63 for much the same reasons as for K^+. Additionally, for Na^+ numerous minor states are observed at low contour levels, which could be an artifact of SDS membrane extraction. We stress, though, that the major state observed in SDS back-exchanged spectra overlays very well with the spectrum obtained without SDS treatment. This issue is not observed in K^+.

Supplementary note 2: ^{15}N relaxation studies of NaK

^{15}N R_2 data collected for NaK in 100 mM K^+ and 600 mM Na^+ are shown plotted against the NaK sequence in Fig. S2. It is clear from the plots in Fig. 2 and Fig. S2 that there is a marked increase in R_1 and decreases in the NOE and R_2 at the ends of the M1 (~18-23) and M2 (~100-110) helices in both salt conditions; this is commonly observed for proteins and is indicative of fraying at the ends of helices. It is also evident that, in general, the measured R_2 values are higher for the Na^+ sample. This is likely due to differences in bicelle size and/or solution viscosity arising from differences in the protein:lipid ratio used for reconstitution (1:60 for K^+, 1:95 for Na^+). Apparent values of the global rotational correlation time (τ_c) were calculated for residues with NOE values >0.65 and ~average R_2/R_1 values, as these residues are unlikely to undergo significant internal dynamics.[1,2] Residue-specific values of τ_c were estimated from the R_2/R_1 ratio for qualifying residues, and then averaged to obtain a global estimate for τ_c. There is a small but significant difference between the K^+ sample, $\tau_c = 43 \pm 2$ ns, and the Na^+ sample, $\tau_c = 47 \pm 2$ ns.

The trend observed in the SF for R_1 and NOE data is generally maintained for R_2, although direct comparison between K^+ and Na^+ conditions is hampered by the aforementioned offset. R_2 values in K^+ for D66 and N68 are elevated relative to the values observed for G67, G69, and S70, whereas R_2 values are generally consistent for residues 66-70 in Na^+. This could be indicative of slower internal dynamics contributing to R_2 for D66 and N68 in K^+.

Supplementary note 3: Effect of ionic strength on observed chemical shifts

In principle, the solution ionic strength (which differed between 100 mM K^+ and 600 mM Na^+ conditions) can influence the NMR chemical shifts of solvent-exposed residues in M2. However, perturbations are observed for backbone nuclei protected by bulky hydrophobic side chains from solvent exposure, some residues that face away from the channel pore, and for $C\alpha$ and L/V $C\delta/C\gamma$ methyl groups (nuclei where chemical shifts are not strongly influenced by through-space, electrostatic effects). Thus, the observed CSPs most likely arise from a conformational change and not simply the difference in ionic strength.

^1H-^{15}N TROSY-HSQC spectra were collected for a U-^2H,^{15}N NaK sample in 100 mM K^+ and 600 mM K^+ to test the extent to which differences in ionic strength affect amide chemical shifts in NaK (Fig. S4). Clearly, the CSPs observed in 100 mM K^+ vs. 600 mM K^+ are in general smaller than the CSPs observed in 100 mM K^+ vs. 600 mM Na^+. The only significant CSPs observed in 100 mM K^+ vs. 600 mM K^+ occur in the SF, but are much smaller than the CSPs observed in the SF between 100 mM K^+ vs. 600 mM Na^+. Thus we are confident that the large CSPs observed in the SF and on M2 (Fig. 3) are the result of a conformational change, rather than solely due to the difference in ionic strength between Na^+- and K^+-bound samples.

a

b

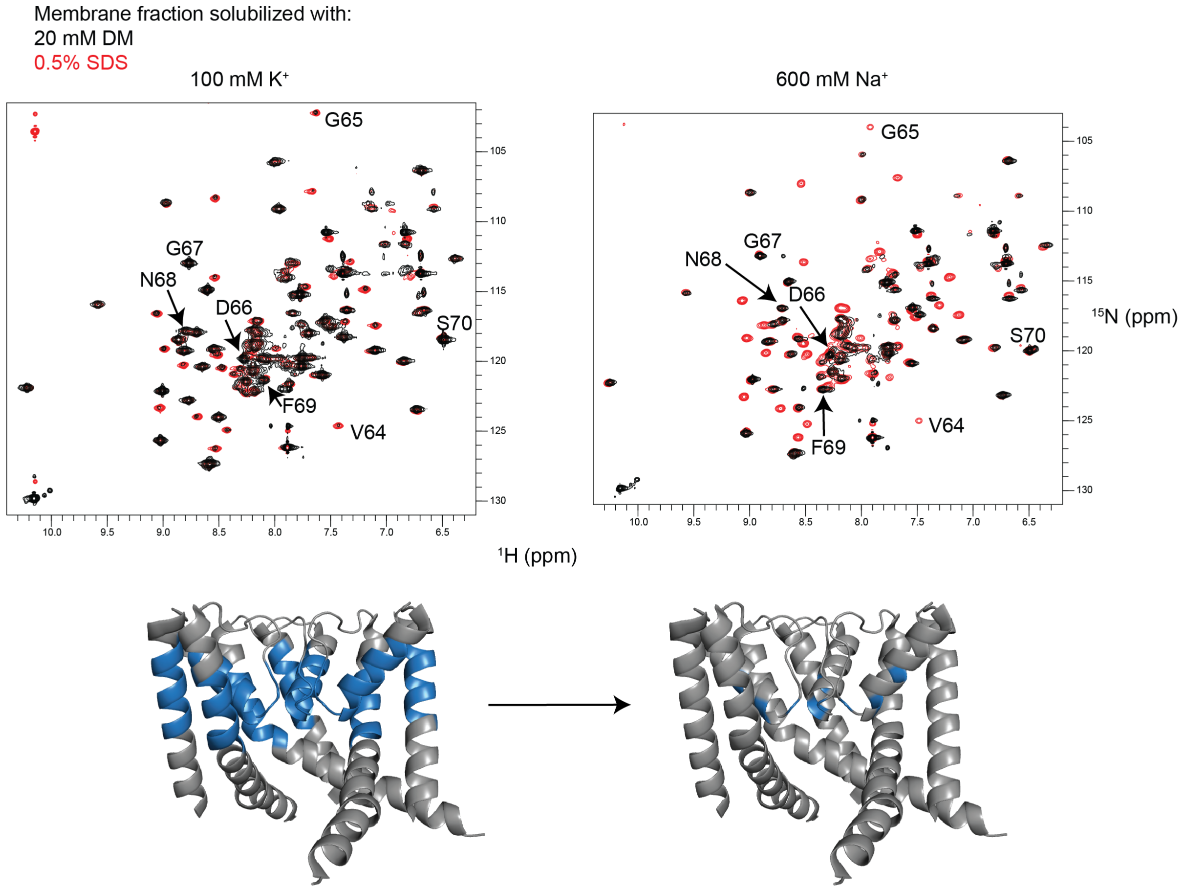

Supplementary Figure 1. Back-exchange of amide protons increases when the membrane fraction is solubilized in 0.5% SDS. a Overlay of ^1H-^{15}N TROSY-HSQC spectra of NaK in 100 mM K^+ (left) and 600 mM Na^+ (right), where the membrane fraction was solubilized with 20 mM DM (black) or 0.5% SDS (red). All spectra were obtained with NaK reconsituted into DMPC:DHPC bicelles (q ~ 0.33). Residues in the SF are indicated; note that V64 and G65 are poorly back-exchanged without SDS membrane extraction. The sidechain peak for W19 is aliased in the back-exchanged spectra. **b** SDS solubilization promoted back-exchange in NaK. On the left, regions with poor back exchange are colored in blue. On the right, residues that remain without assignment after SDS solubilzation are colored blue. Residues turning from blue to gray increase in intensity after solubilization with SDS, indicating increased back-exchange.

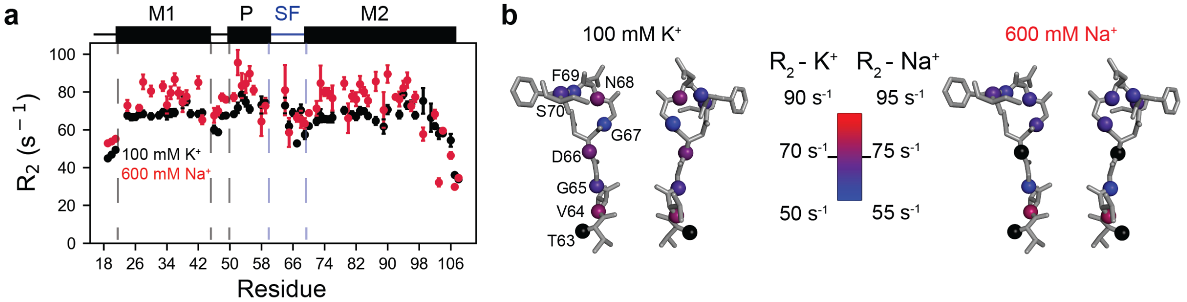

Supplementary Figure 2. ^{15}N R_2 values vary across NaK SF. a Backbone amide ^{15}N R_2 values recorded at 750 MHz for NaK in 100 mM K^+ (black) and 600 mM Na^+ (red). R_2 values are calculated from R_1 and $R_1\rho$ as described in the Methods. Error bars were calculated as the SD of 500 Monte Carlo simulations, as described in detail in the Methods. Secondary structure elements are shown above, where helices are rectangles and loops are lines. The boundaries between elements are based on the 3E8H crystal structure. Residues 63-70 are indicated by the blue lines and are shown as sticks in B. **b** Relaxation data plotted on the NaK SF (showing 2 of 4 opposing subunits). Backbone N atoms are shown as spheres and are colored according to the scale indicated for each parameter. Residues for which data are unavailable due to resonance overlap or lack of assignment are colored black.

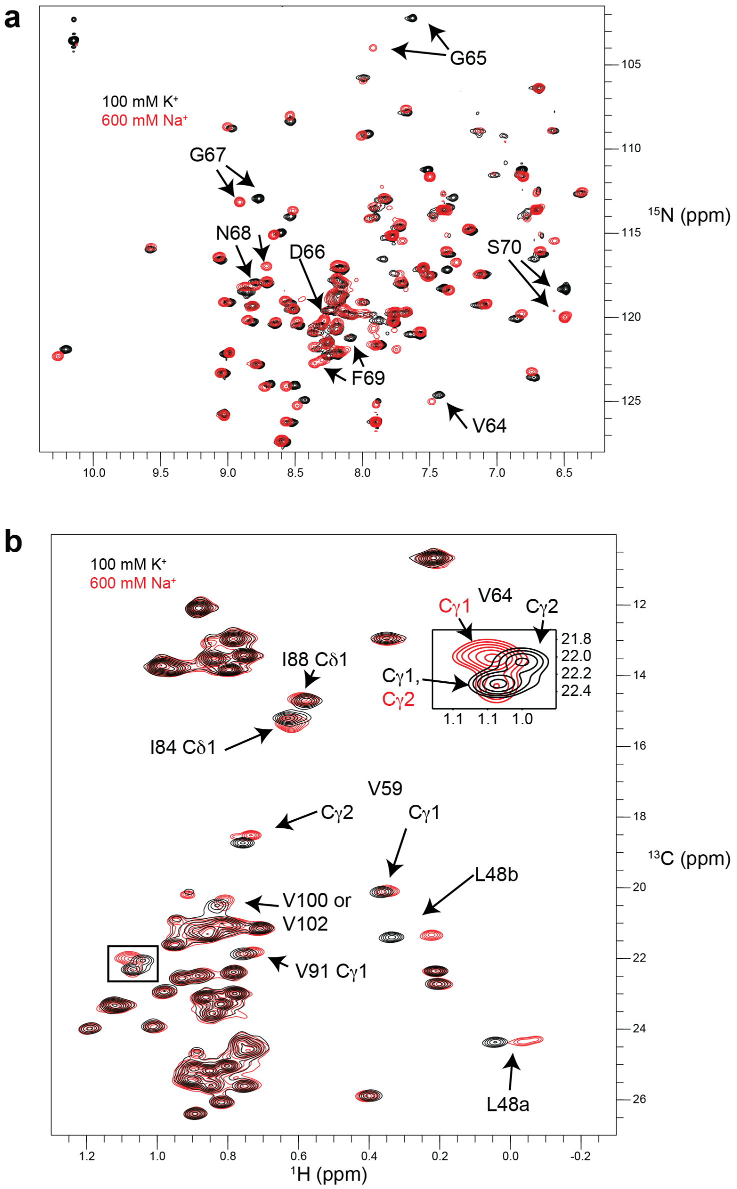

Supplementary Figure 3. Overlay of NaK spectra in 100 mM K^+ vs. 600 mM Na^+. a TROSY-HSQC spectra of NaK in the indicated ionic condition. Residues in the SF that undergo significant chemical shift perturbations are indicated. Note that the W19 sidechain amide peak is aliased in these spectra. **b** ^{1}H-^{13}C HMQC spectra of ILV-labeled NaK in the indicated ionic conditions. Peaks near the SF that shift between conditions are labeled. Methyl resonances that are stereospecifically assigned are labeled according to the PDB labeling for 3E8H, while resonances without stereospecfic assignments are labeled a or b. Resonances for V64 are expanded in the inset and labels are color-coded red for Na^+ and black for K^+.

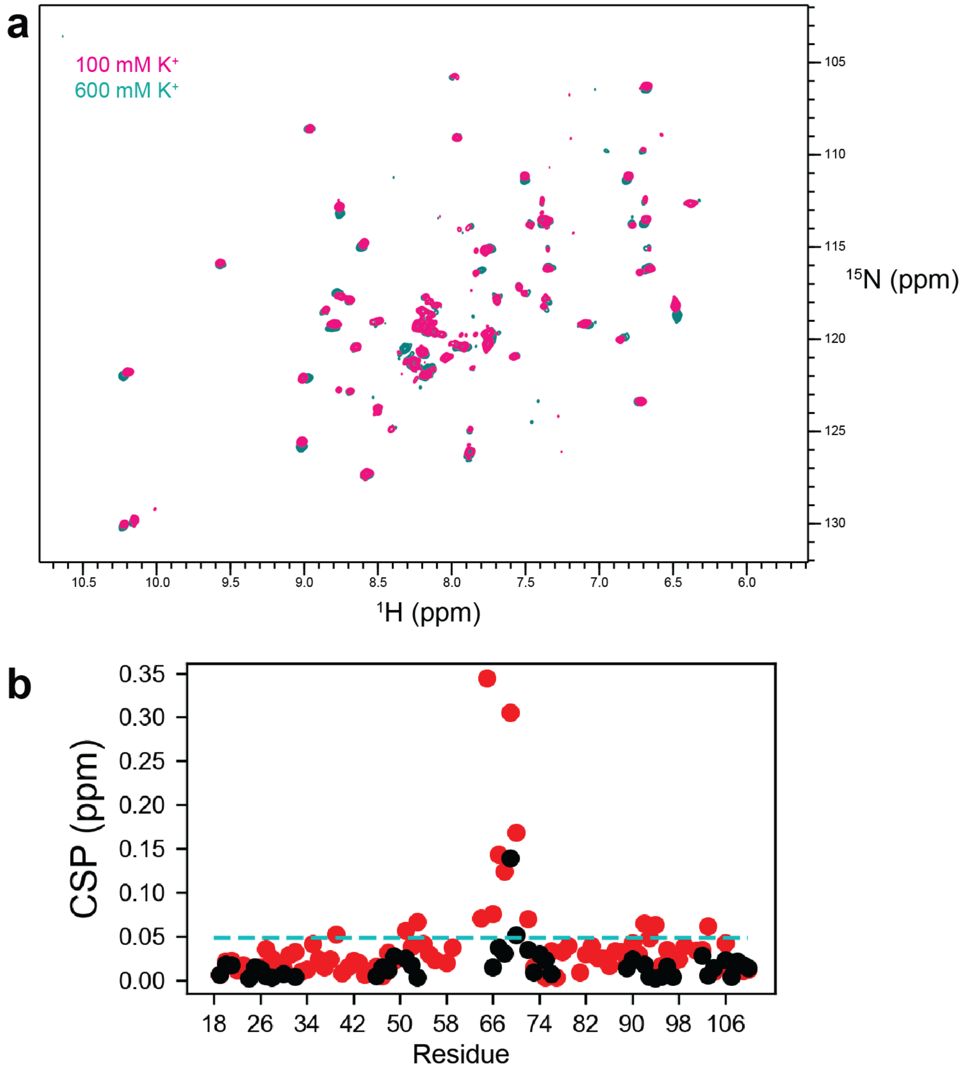

Supplementary Figure 4. Ionic strength has a minimal effect on H-N chemical shifts. (**a**) TROSY-HSQC spectra of NaK in 100 mM (magenta) and 600 mM K^+ (teal). (**b**) Chemical shift perturbations (CSPs) calculated for 100 mM vs. 600 mM K+ (black) and for 100 mM K^+ vs. 600 mM Na^+ (red, same as Fig. 3). The cyan line is plotted at 1.5σ above the 10% trimmed mean for the case of 100 mM K^+ vs. 600 mM Na^+ (as in Fig. 3). CSPs due to ionic strength alone are consistently significantly smaller than the CSPs observed between K^+ and Na^+.

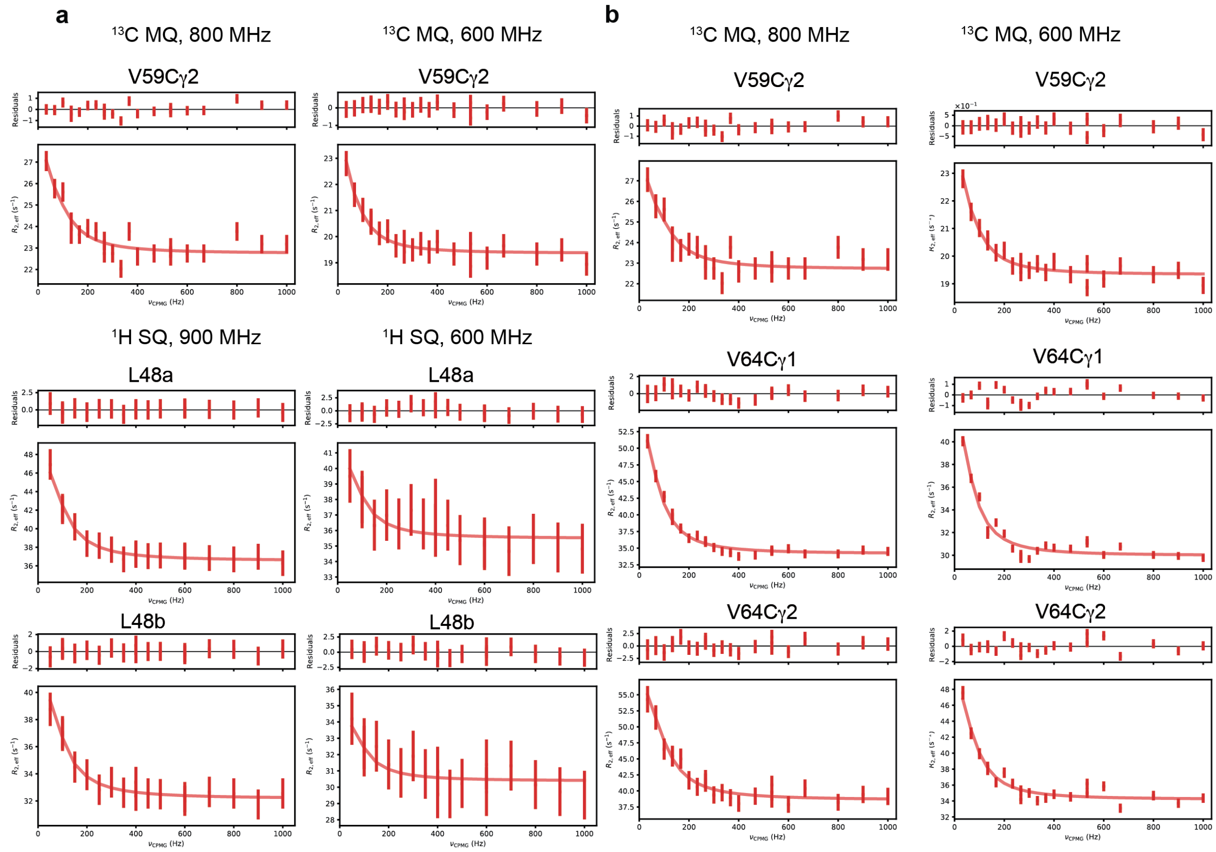

Supplementary Figure 5. Fit results for global fitting of CPMG data. a Fit results for simultaneous fitting of V59 ^{13}C MQ and L48 ^{1}H SQ CPMG data. The extracted value of k_{ex} = 356 ± 67 s^{-1}. **b** Fit results for simultaneous fitting of ^{13}C MQ data for V59 and V64. The extracted value of k_{ex} = 390 ± 47 s^{-1}.

REFERENCES:

1. Jarymowycz, V. A. & Stone, M. J. Fast Time Scale Dynamics of Protein Backbones: NMR Relaxation Methods, Applications, and Functional Consequences. *Chem. Rev.* **106**, 1624–1671 (2006).
2. Kay, L. E., Torchia, D. A. & Bax, A. Backbone dynamics of proteins as studied by nitrogen-15 inverse detected heteronuclear NMR spectroscopy: application to staphylococcal nuclease. *Biochemistry* **28**, 8972–8979 (1989).
