# Supplementary material for: Ion-dependent structure, dynamics, and allosteric coupling in a non-selective cation channel
Source: Nat Commun. 2021 Oct 28;12:6225. doi: 10.1038/s41467-021-26538-8 (PMC8553846; doi:10.1038/s41467-021-26538-8)
Supplement: Supplementary file 2 — Description of Additional Supplementary Files [file 41467_2021_26538_MOESM2_ESM.pdf]

Description of Additional Supplementary Files

File name: Supplementary Data 1

Description: 15N relaxation parameters for NaKΔ18 in 100 mM K+ and 600 mM Na+. Same data as in Fig. 2. Errors represent the SD of 500 Monte Carlo simulations, as described in the Methods. Residues for which data are not available due to resonance overlap or lack of assignment are denoted n.d.

File name: Supplementary Data 2

Description: Chemical shift perturbation (CSP) and Δδ values for NaKΔ18 in 100 mM K+ and 600 mM Na+. Same data as in Fig. 3. CSPs and Δδ values were calculated as described in the Methods. Residues for which data are not available due to resonance overlap or lack of assignment.

File name: Supplementary Data 3

Description: 13C Multiple Quantum (MQ) Rex values for NaKΔ18 in 100 mM K+ and 600 mM Na+. Same data as in Fig. 6. Rex was calculated by subtracting the first and last points of the dispersion curves, as described in the Methods. The error in Rex is the SD obtained by standard error propagation.
