# Supplementary material for: Ion-dependent structure, dynamics, and allosteric coupling in a non-selective cation channel
Source: Nat Commun. 2021 Oct 28;12:6225. doi: 10.1038/s41467-021-26538-8 (PMC8553846; doi:10.1038/s41467-021-26538-8)
Supplement: Supplementary file 6 — Reporting Summary [file 41467_2021_26538_MOESM6_ESM.pdf]

nature portfolio

Corresponding author(s): Katherine Henzler-Wildman
Last updated by author(s): Sep 28, 2021

Reporting Summary

Nature Portfolio wishes to improve the reproducibility of the work that we publish. This form provides structure for consistency and transparency in reporting. For further information on Nature Portfolio policies, see our Editorial Policies and the Editorial Policy Checklist.

Statistics

For all statistical analyses, confirm that the following items are present in the figure legend, table legend, main text, or Methods section.

n/a	Confirmed	
☐	☒	The exact sample size (*n*) for each experimental group/condition, given as a discrete number and unit of measurement
☐	☒	A statement on whether measurements were taken from distinct samples or whether the same sample was measured repeatedly
☒	☐	The statistical test(s) used AND whether they are one- or two-sided<br>*Only common tests should be described solely by name; describe more complex techniques in the Methods section.*
☒	☐	A description of all covariates tested
☒	☐	A description of any assumptions or corrections, such as tests of normality and adjustment for multiple comparisons
☐	☒	A full description of the statistical parameters including central tendency (e.g. means) or other basic estimates (e.g. regression coefficient) AND variation (e.g. standard deviation) or associated estimates of uncertainty (e.g. confidence intervals)
☒	☐	For null hypothesis testing, the test statistic (e.g. *F*, *t*, *r*) with confidence intervals, effect sizes, degrees of freedom and *P* value noted<br>*Give P values as exact values whenever suitable.*
☒	☐	For Bayesian analysis, information on the choice of priors and Markov chain Monte Carlo settings
☒	☐	For hierarchical and complex designs, identification of the appropriate level for tests and full reporting of outcomes
☒	☐	Estimates of effect sizes (e.g. Cohen's *d*, Pearson's *r*), indicating how they were calculated

Our web collection on statistics for biologists contains articles on many of the points above.

Software and code

Policy information about availability of computer code

Data collection	NMR data collected on Bruker and Varian spectrometers was collected using Topspin version 3.5pl7 and vnmrj version 4.2 respectively.
Data analysis	NMR data was processed using nmrPipe version 9.0 and and spectra were visualized using ccpNmr analysis version 2.4.2. Reconstruction of non-uniformly sampled NMR data was performed using the SMILE algorithm (version 1.1) implemented in nmrPipe. R1 and R1rho values were determined by extracting peak heights using ccpNmr analysis and fitting to a single exponential decay with a home-written script using the scipy.optimize.curve_fit library. The code can be made available upon request. The published software MAGMA (version 1.2.3) was used as part of the methyl resonance assignment procedure. Methyl CPMG data was fit using the published ChemEx software (version 2019.1.0).

For manuscripts utilizing custom algorithms or software that are central to the research but not yet described in published literature, software must be made available to editors and reviewers. We strongly encourage code deposition in a community repository (e.g. GitHub). See the Nature Portfolio guidelines for submitting code & software for further information.

Data

Policy information about availability of data

All manuscripts must include a data availability statement. This statement should provide the following information, where applicable:

- Accession codes, unique identifiers, or web links for publicly available datasets
- A description of any restrictions on data availability
- For clinical datasets or third party data, please ensure that the statement adheres to our policy

Backbone amide and ILV methyl chemical shifts in K+ and Na+ are available in BMRB under deposition numbers 51063 and 51064 respectively. Raw NMR data is deposited in BMRBig with deposition IDs bmrbig25 and bmrbig26 for K+ and Na+ data respectively. Chemical shift perturbation values, 15N relaxation parameters,

and CPMG Rex values are provided in the Supplementary Information. This study utilized PDB accession codes 3E8H, 3E86, 6CPV, 6V8Y, 3LDC, 1K4C, and 3J5Q.

Field-specific reporting

Please select the one below that is the best fit for your research. If you are not sure, read the appropriate sections before making your selection.

☒ Life sciences ☐ Behavioural & social sciences ☐ Ecological, evolutionary & environmental sciences

For a reference copy of the document with all sections, see nature.com/documents/nr-reporting-summary-flat.pdf

Life sciences study design

All studies must disclose on these points even when the disclosure is negative.

Sample size	One sample for each condition (K+ and Na+) was used to obtain backbone chemical shifts. Separate samples, again one for each condition, were used for 15N relaxation experiments. Methyl NOESY and CPMG data in K+ were collected on a single sample, while NOESY and CPMG experiments in Na+ were collected on two separate samples. No statistical methods were used to determine the sample size. Experiments were not repeated on additional samples because these experiments are costly in terms of time and materials. Peak positions agree very well between independently prepared samples, giving us confidence that the sample size is sufficient.
Data exclusions	No data was excluded
Replication	All 3D experiments to obtain backbone chemical shifts were performed once, on one sample each in K+ and Na+. 15N relaxation experiments were performed once on one sample each for K+ and Na+. Methyl CPMG and NOESY experiments were collected once, on one sample for K+ and using two samples for Na+. Errors in R2eff for CPMG experiments were calculated as the standard deviation from 3 replicated data points.
Randomization	This study did not require randomization. This study does not involve comparisons between different cohorts hence randomization is not applicable.
Blinding	Blinding is not possible for this study because the researchers must know the details of each sample in order to interpret the data.

Reporting for specific materials, systems and methods

We require information from authors about some types of materials, experimental systems and methods used in many studies. Here, indicate whether each material, system or method listed is relevant to your study. If you are not sure if a list item applies to your research, read the appropriate section before selecting a response.

Materials & experimental systems

n/a	Involved in the study	
☒	☐	Antibodies
☒	☐	Eukaryotic cell lines
☒	☐	Palaeontology and archaeology
☒	☐	Animals and other organisms
☒	☐	Human research participants
☒	☐	Clinical data
☒	☐	Dual use research of concern

Methods

n/a	Involved in the study	
☒	☐	ChIP-seq
☒	☐	Flow cytometry
☒	☐	MRI-based neuroimaging
